# Editorial: High-Frequency Oscillations in the Hippocampus as Biomarkers of Pathology and Healthy Brain Function

**DOI:** 10.3389/fnhum.2021.763881

**Published:** 2021-09-29

**Authors:** Johannes Sarnthein, Julia Jacobs, Maeike Zijlmans

**Affiliations:** ^1^University Hospital and University of Zurich, Zurich, Switzerland; ^2^Alberta Children's Research Institute, University of Calgary, Calgary, AB, Canada; ^3^University Medical Center Utrecht, Utrecht, Netherlands; ^4^Stichting Epilepsie Instellingen Nederland (SEIN), Heemstede, Netherlands

**Keywords:** high frequency oscillation, epilepsy, memory, hippocampus, cognition, ripple, fast ripple

Functional and anatomical aspects of the hippocampus are unique compared to other brain regions. Brain signals recorded from this structure are indicators for function especially memory, but also diseases like epilepsy or dementia. The hippocampus was the first structure in which High frequency oscillations (HFO > 80 Hz) were discovered. From recent research it is also the place where HFO are most abundant. This discovery raises an important question: are these oscillations reflecting function or pathology?

A clear link between HFO and pathology has been found for patients with epilepsy. Animal models suggest that epileptic HFO only occur in rodents that develop spontaneous seizures after induced status epilepticus. If HFOs are detected in the tissue during the invasive pre-surgical examination in patients with refractory epilepsy, the resection of this tissue is a specific predictor for postoperative seizure freedom. While first identified over the hippocampus, the validity of HFO as biomarker for epilepsy extends to all other brain lobes.

Similarly, there is no question that HFO contribute to important physiological functions in the hippocampus. Physiological HFO were first identified in rat hippocampus, where ripples (80–250 Hz) contribute to spatial processing. The visual cortex and the somatosensory cortex abound with HFO that seem unrelated to the epileptogenicity of the tissue—they mask possible pathological HFO and render HFO analysis not applicable in these brain areas. HFO are certainly a part of the repertoire of oscillations in the healthy cortex.

Therefore, in the MTL, both pathological and healthy HFO are of high scientific interest and we have to pose the following question: Is the co-existence of physiological and epileptic HFO a confounding factor for using HFO in diagnostics? Can the overlap in frequencies also be an opportunity to learn about both function and pathology in the hippocampus? We therefore designed a Research Topic with a specific focus on these questions. In the following papers you will find a wide range of methods and questions all aimed to discuss “High-Frequency Oscillations in the hippocampus as biomarkers of pathology and in healthy brain function.”

Contributions in this special issue span from improvements in the methodology of analyzing HFO to investigating the link between HFO and function/pathology. At this point, there is no agreement on the actual definition and mechanisms of HFO. These questions are first addressed from a signal processing perspective (Thomschewski et al.) and from a physiological perspective to advance our understanding on a microscopic level (Naggar et al.; Weiss et al.). Naggar et al. showed that in rat brains hippocampal slices HFOs had the highest amplitude over the CA3c region. Weiss et al. showed that epileptiform ripples occurred mostly during the on-off state transition of hippocampal slow waves. Additionally, one of the contributions reviews how different EEG frequencies have been linked to memory and come to the conclusion that t higher frequencies appear most interesting to study memory functioning (Arski et al.).

Several contributions in this collection aim to shed light on the differences between physiological and epileptic HFO. In this effort two principal approached were used. First, studies analyzed changed in HFO occurrence and rate during cognitive tasks. Second, HFO were not analyzed as stand-alone events but in their occurrence with other markers like epileptic spikes or sleep spindles.

Cimbalnik et al. show in 24 patients with bilateral stereo-EEG implantations that cognitive tasks reduced epileptiform activity in the diseased hippocampus. At the same time, brain activity in the healthy hippocampus shifted toward higher frequencies. With machine learning they created a predictive model for the diseased hippocampus based on HFOs, connectivity and spikes.

A set of studies focused on comparing distribution and changes in subsets of ripples, namely in isolated ripples (maybe physiological), spindle-ripples (likely physiological) and epileptic spikes coupled ripples. Bruder et al. focused on describing the occurrence of sleep-spindle coupled ripples. The latter are believed to be a subset of ripples, linked to physiological task. In the present study they were identified most frequently but not limited to the hippocampal structure. As second study investigated how cognitive tasks modulate the above described ripples subtypes (Lachner-Piza et al.). While cognitive tasks reduced the number of isolated ripples in the diseased hippocampus, no effect was observed for ripples co-occurring with spikes. Most importantly, authors found a positive correlation between performance improvement and spindle-ripple rates in a spatial navigation task. This finding suggests that spindle ripples actually select a physiological subpopulation of all HFO. Moreover, that rates of physiological HFO might allow us to measure function.

Interestingly, a similar correlation could not be found in other studies, which may relate to the specific definition of what is an epileptic HFO or a physiological HFO. Thomschewski et al. found no relation between performance of memory tasks and number of automatically detected HFOs. Agudelo Valencia et al. also showed no relation between ripples (either normal or prolonged) and IQ scores. As a further null-finding, Boran et al. did not find an effect of cognitive tasks on HFO rates. More specifically fast-ripples co-occurring with ripples were not altered during the task. This finding is reassuring, as ripples with fast ripples can be used for pre-surgical evaluations and seem to occur independent of behavioral changes.

In keeping with the tradition of HFO as biomarker for epilepsy, several contributions in the collection focus on the relation of HFO with the underlying pathology and epileptic activity. In the past several studies focused on the question whether HFO are linked to abnormal “lesional” brain tissue in general more specifically reflect the epileptic potential of this tissue. Agudelo Valenca et al. confirmed that high HFO rates occur in brain regions with hippocampal sclerosis. Interestingly the same was not true for areas which only showed atrophy. In the study of Schönberger et al. fast ripples, but not ripples or spikes, could predict the epileptogenic focus in case of dual pathology (lesions and hippocampal sclerosis). Boran et al. reported a higher HFO rate in the seizure onset zone not only during deep sleep but even during wakefulness while performing cognitive tasks. These studies can be viewed as additional evidence that HFO are not just reflecting general anatomical changes but are more specific for epilepsy. One contribution reports that HFO might even serve as predictors of imminent seizures in 10 out of 27 patients, mostly with temporal lobe epilepsy (Scott et al.).

Neuroscientists and epileptologists have long known that physiological function and epileptic activity co-exist in the human brain even within small substructures like the hippocampus. The present collection of articles focusses on investigating the value of HFO in this triangle between different cognitive functions and brain pathology. Figure 1 summarizes the different interactions investigated. As expected, this summary cannot give a final answer to all open questions and some findings are slightly contradictory. If anything, these papers confirm the complex relations between HFOs and physiological functioning as well as with pathology and epileptogenicity.

**Figure 1:**
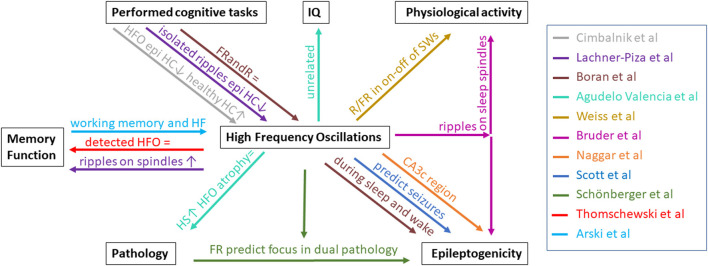
Overview of relationships between HFO and pathology, memory function, performed cognitive tasks, IQ, physiological activity, and epileptogenicity described by the papers in this Research Topic. Each paper is represented by a color. HS, hippocampal sclerosis; HC, hippocampus; epi, epileptic; FRandR, fast ripples combined with ripples; R, ripple; FR, fast ripple; SW, slow waves; ↑, increase/positive relation; =, no effect; ↓, decrease/negative relation.

Overall we have to keep in mind that the term HFO simply describes a frequency band and not all oscillations in this frequency serve the same purpose. Thereby, this Research Topic exemplifies the current research directions in the fields of HFO in healthy brain function and in epilepsy. It can be seen as guide suggesting new methods and pathways to separating physiology and pathology within the epileptic hippocampus.

## Author Contributions

All authors listed have made a substantial, direct and intellectual contribution to the work, and approved it for publication.

## Funding

We acknowledge the ERC starting grant 803880 to MZ and the SNSF grant 176222 to JS.

## Conflict of Interest

The authors declare that the research was conducted in the absence of any commercial or financial relationships that could be construed as a potential conflict of interest.

## Publisher's Note

All claims expressed in this article are solely those of the authors and do not necessarily represent those of their affiliated organizations, or those of the publisher, the editors and the reviewers. Any product that may be evaluated in this article, or claim that may be made by its manufacturer, is not guaranteed or endorsed by the publisher.

